# Sphinkeeper™ for faecal incontinence: a preliminary report

**DOI:** 10.1111/codi.14801

**Published:** 2019-08-16

**Authors:** M. La Torre, G. Lisi, G. Milito, M. Campanelli, I. Clementi

**Affiliations:** ^1^ Department of Surgery Policlinico Umberto Primo Sapienza University Rome Italy; ^2^ Department of General Surgery Sant'Eugenio Hospital Rome Italy; ^3^ Department of General Surgery Clinica Valle Giulia Rome Italy; ^4^ Department of General Surgery University of Modena and Reggio Emilia Modena Italy; ^5^ Department of Emergency Policlinico Umberto Primo Sapienza University Rome Italy

**Keywords:** faecal incontinence, sphincter lesion, artificial anal sphincter, endoanal ultrasound, SphinKeeper™

## Abstract

**Aim:**

A new artificial anal sphincter placed into the intersphincteric space, SphinKeeper™, has recently been proposed to improve outcomes in the treatment of faecal incontinence (FI). We report our preliminary results with short‐term follow‐up, comparing preoperative and postoperative data after implant of SphinKeeper™ in patients suffering from FI.

**Methods:**

All patients older than 18 years were included with FI of at least 6 months, incontinence episodes occurring more than once a week and resistance to other conservative treatments. Anorectal manometry, endoanal ultrasound, Cleveland Clinic FI Score, FI Quality of Life score and total number of episodes of FI per week were recorded preoperatively and at the end of the 6‐month follow‐up period.

**Results:**

Thirteen consecutive patients were treated with SphinKeeper™. No intra‐operative nor postoperative complications were reported. Two cases of prosthesis extrusion occurred, and in one case an anterior dislocation was detected. Maximum resting pressure, total number of episodes of FI per week and Cleveland Clinic FI Score were improved after 6 months (*P *<* *0.05).

**Conclusions:**

SphinKeeper™ could be a minimally invasive procedure for FI with good postoperative outcomes. If these results are confirmed by studies with more patients and longer follow‐up, it could be a first‐line approach in FI.

**What does this paper add to the literature?**

This study provides evidence of the safety, feasibility and good outcome of SphinKeeper™ in the treatment of faecal incontinence, and the technique compares well with other more invasive techniques. It is also the first article on SphinKeeper™ with a 6‐month follow‐up and includes patients with a sphincter injury.

## Introduction

Faecal incontinence (FI) is a frequent and complex condition with a significant impact on psychological well‐being and quality of life and is associated with high social and health costs. Its prevalence in the USA, comparable in men and women, is estimated at around 10% of the population over 21 years (2%–20%) and increases with age 1, 2. Several treatments are available for FI, from conservative such as pelvic floor exercise, biofeedback and electrical stimulation, each with a high failure rate, less invasive procedures such as bulking agents, sacral and tibial nerve stimulation and more invasive such as graciloplasty or dynamic graciloplasty, sphincteroplasty and artificial magnetic sphincter. Unfortunately, the results of all of these techniques are variable 3. A new artificial anal sphincter inserted into the intersphincteric space, SphinKeeper™, has recently been described by Ratto *et al*. 4 to improve results in the treatment of FI. We report our preliminary results with a short‐term follow‐up, comparing preoperative and postoperative data before and after the implant of SphinKeeper^™^ in patients with FI.

## Method

SphinKeeper™ prostheses (THD SpA, Correggio, Italy) are made of Hyexpan (polyacrylonitrile). In the dehydrated state they are thin solid cylinders (length 29 mm, diameter 3 mm), which change shape and size [become shorter (23 mm) and wider (diameter 7 mm)] and physical properties (softer, with shape memory) within 48 h of contact with fluid. The study was approved by the local ethics committee and all procedures were performed by the same two surgeons (MLT and IC) at Villa Tiberia Hospital, Rome, Italy, with the Department of Surgery, Sapienza University of Rome, Italy.

We included all patients over the age of 18 years; with FI (incontinence to liquid and/or solid stools) that had started at least 6 months before; episodes of FI that occurred more than once a week and resistant to conservative treatments (e.g. stool bulking and/or constipating agents); and endoanal ultrasound (EAUS) assessment showing intact anal sphincters or a sphincter injury [internal anal sphincter (IAS), external anal sphincter (EAS) or both]. The exclusion criteria were malignant neoplasms, unknown cause of rectal bleeding, congenital anorectal malformations, inflammatory bowel disease, sepsis, obstructive defaecation syndrome, neurological disease and coagulation disorders.

All patients were evaluated before surgery with a complete proctological examination including previous proctological history, anoscopy and colonoscopy, if necessary. Preoperative, intra‐operative and postoperative data were recorded on our prospective database: anorectal manometry (AM) with the Anopress™ device (THD SpA), EAUS with the Aqua Vu™ (USB‐12 MHz high resolution endocavity probe; Laborie®, Mississauga, ON, Canada), the Cleveland Clinical Faecal Incontinence Score (CCFIS) 5 and the Faecal Incontinence Quality of Life (FIQL) score 6. Also the total number of FI episodes per week was recorded. After a complete explanation by a member of the surgical team, informed consent was signed by all patients.

All patients were instructed to avoid constipation and hard stools (which can lead to early displacement of the prosthesis), so behavioural changes were advised and dietary fibre supplements were prescribed after surgery (e.g. stool bulking). As described by Ratto *et al*. 4, the patients had bowel preparation with two 120 ml docusate sodium enemas, 12 and 2 h before the treatment. Antibiotic prophylaxis of 1 g of intravenous cefazolin and 500 mg of intravenous metronidazole was given. The prostheses were checked with EAUS at 1 week, 1 month and 6 months after surgery. The primary end‐point was to evaluate the safety of the procedure in terms of intra‐operative and postoperative adverse events and complications. The secondary end‐point was to assess the effectiveness of SphinKeeper™ injection in terms of improvement of FI, manometric parameters and quality of life. CCFISs were calculated with a continence diary kept by all patients and the FIQL questionnaires were compiled at the end of the follow‐up.

### Statistical analysis

spss® version 17.0 (IBM, Armonk, New York, USA) was used for the statistical analysis. Data were expressed as median with range. The Mann–Whitney *U* test was used to analyse the quantitative variables and *P *<* *0.05 was considered statistically significant.

### Surgical technique

SphinKeeper™ prostheses were implanted as described previously 4 under general or spinal anaesthesia in the lithotomy position; 10 2‐mm skin incisions were made 2–3 cm from the anus, at 1, 2, 3, 4, 5, 7, 8, 9, 10, 11 o'clock positions, equidistant from each other. A careful, blind dissection into the intersphincteric space was performed using Kelly forceps, and once this tunnel was created the delivery system was placed until it reached the upper intersphincteric space. Implantation was checked carefully using intra‐operative EAUS confirming the correct position of the implants in the intersphincteric space, with the apex of the implant at the upper edge of the puborectalis muscle. After implantation EAUS confirmed the location of the prosthesis, which appears as a hyperechoic dot with a hypoechoic shadow behind it. The same procedure was repeated for all 10 prostheses around the entire circumference of the IAS. Finally, skin wounds were sutured with absorbable sutures. All patients were strongly advised to observe bed rest or to move slowly from bed to chair for 48 h to minimize early prosthesis dislocation.

## Results

Between December 2016 and February 2018, 13 consecutive patients (10 women and three men) affected by FI were treated with SphinKeeper™ prostheses. Baseline, preoperative and postoperative data of each patient are shown in Table 1.

**Table 1:** Preoperative and postoperative data.

Patient (M/F)	Past history	Clinical examination	EAUS (degree of injury)	AM resting (mmHg pre/post)	AM squeeze (mmHg pre/post)	CCFIS (pre/post)	No. of FI per week (pre/post)
1 (M)	Fistulotomy, sphincteroplasty	FI to gas and liquid stool	EAS/IAS lesion (65°)	11/26	65/72	13/9	8/2
2 (M)	Haemorrhoidectomy	FI to gas and liquid stool	IAS lesion (70°)	25/38	98/105	12/6	6/2
3 (F)	Forceps delivery, sphincteroplasty	FI to gas and liquid stool	EAS lesion (82°)	27/35	97/100	11/7	6/2
4 (F)	Fistulectomy, sphincteroplasty	FI to gas and liquid stool	EAS/IAS lesion (30°)	22/31	89/91	13/10	5/1
5 (F)	Forceps delivery, multiparity	FI to gas and liquid stool	EAS/IAS lesion (55°)	28/33	80/86	14/10	5/1
6 (F)	Forceps delivery, multiparity	FI to gas and liquid stool	IAS lesion (20°)	26/39	105/106	10/6	3/1
7 (F)	Multiparity	FI to gas and liquid stool	EAS/IAS lesion (15°)	18/29	83/90	14/10	3/1
8 (F)	Radiotherapy, LAR	FI to gas and liquid stool	IAS inhomogeneity	20/30	95/96	15/11	4/2
9 (M)	Anal fissure, sphincterotomy	FI to gas and liquid stool	IAS inhomogeneity	19/29	92/98	10/9	3/1
10 (F)	Rectosigmoid resection, ventral rectopexy, Delorme	FI to gas and liquid stool	EAS/IAS inhomogeneity	10/20	30/40	15/11	11/5
11 (F)	Forceps delivery, multiparity	FI to gas and liquid stool	EAS/IAS inhomogeneity	23/35	68/76	11/6	3/1
12 (F)	Anal fissure, sphincterotomy	FI to gas and liquid stool	IAS lesion (57°)	30/41	95/102	13/10	4/2
13 (F)	Ventral rectopexy	FI to gas and liquid stool	EAS/IAS inhomogeneity	18/28	79/92	15/12	6/4

AM, anorectal manometry; CCFIS, Cleveland Clinic Faecal Incontinence Score; EAS, external anal sphincter; EAUS, endoanal ultrasound; F, female; FI, faecal incontinence; IAS, internal anal sphincter; LAR, low anterior resection; M, male.

**Figure 1:**
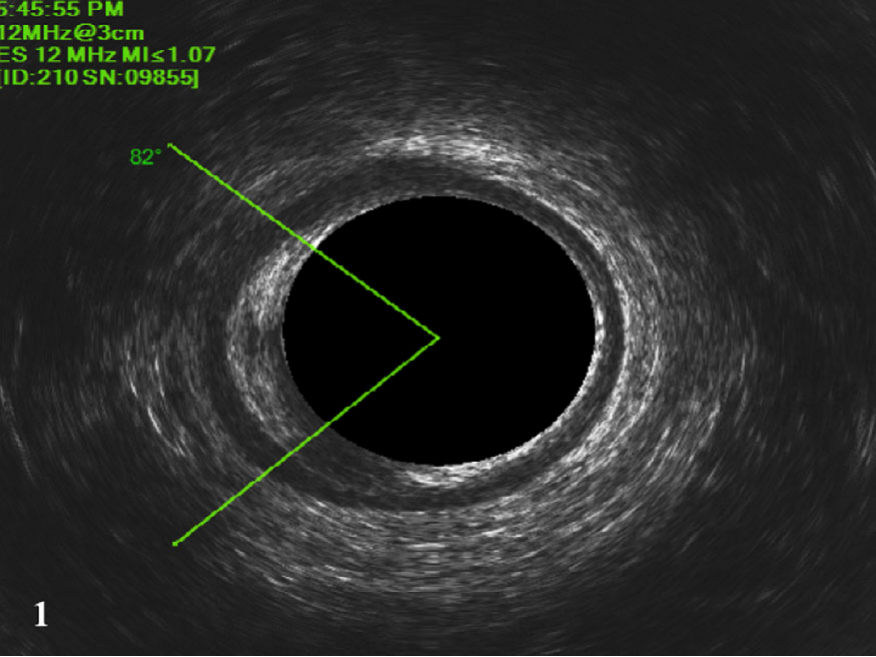
Two‐dimensional endoanal ultrasound showing 82° EAS lesion.

**Figure 2:**
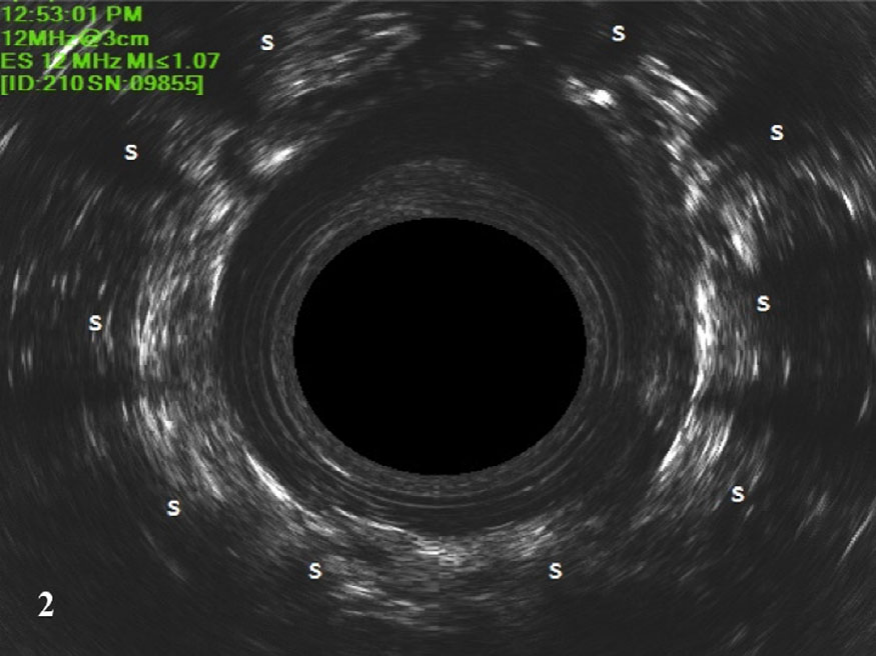
Intra‐operative EUAS showing the prosthesis in the dehydrated state. Implants (s) appear as hyperechogenic images in the intersphincteric space.

**Figure 3:**
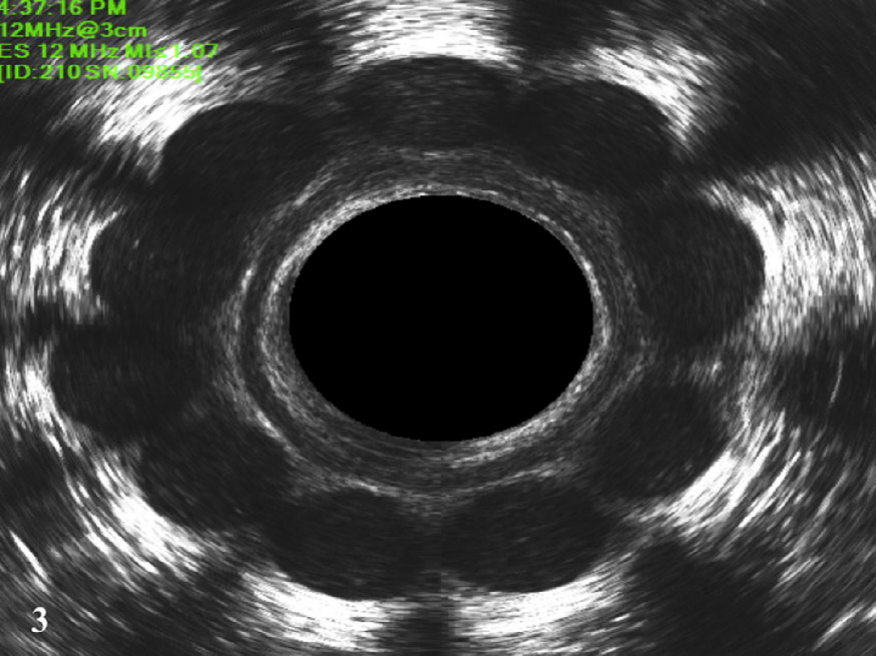
Two‐dimensional EUAS showing the prostheses after 6 months.

The number of prostheses implanted was 10 in all patients with a median operating time for the SphinKeeper™ implant using EAUS guidance of 41 min (range 33–48 min). The present series included patients with intact sphincters, and with IAS and EAS defects. The prostheses were placed in the same position in all patients, regardless of the position of the sphincter injury. No intra‐operative or in‐hospital complications were reported. In this preliminary report, the follow‐up for each patient was 6 months. After a week, by EAUS assessment all the prostheses achieved the final size, even those implanted in scar tissue (Figs 1,2 and 3). No anorectal pain or discomfort occurred during the entire follow‐up period.

Two cases of a prosthesis extrusion occurred 1 month after surgery: in one male patient there was a posterior extrusion and in a female patient an anterior extrusion occurred. Anterior dislocation, defined as a prosthesis not at the same level as other implants, was detected in one female patient 6 months after surgery. The mean and range of preoperative and postoperative AM (reference values: resting pressure range 40–80 mmHg; squeeze pressure range 105–261 mmHg), EAUS, CCFIS and FIQL score are shown in Table 2.

**Table 2:** Preoperative and postoperative data.

	Preoperative	Postoperative	*P*
AM maximum resting pressure (mmHg); mean (range)	21.3 (10–30)	31.84 (20–41)	< 0.05
AM maximum squeeze pressure (mmHg); mean (range)	83 (30–105)	88.53 (40–106)	NS
CCFIS; mean (range)	12.46 (10–15)	8.91 (6–12)	< 0.05
FIQL score			
Lifestyle; mean (range)	2.62 (2.2–3.1)	3.2 (2.9–3.5)	NS
Coping and behaviour; mean (range)	1.97 (1.7–2.2)	2.37 (2–2.6)	NS
Depression and self‐perception; mean (range)	2.96 (2.7–3.2)	3.39 (3.1–3.6)	NS
Embarrassment; mean (range)	2.46 (2–2.8)	3 (2.7–3.4)	NS
Total number of episodes of FI per week	5.38 (2–11)	1.57 (1–5)	< 0.05

AM, anorectal manometry; CCFIS, Cleveland Clinic Faecal Incontinence Score; FI, faecal incontinence; FIQL, Faecal Incontinence Quality of Life; NS, not significant.

## Discussion and conclusions

Various injectable agents have been used to improve FI, although the techniques, the materials and the most useful placement sites are not yet standardized. Moreover, the results are controversial and hard to interpret. A Cochrane review analysed five randomized trials with 382 patients, concluding that all five studies were limited, with methodological bias 7. Hong *et al*. 8 in their systematic review and meta‐analysis of medium‐term outcomes in FI after treatment with injectable materials found that the agents used result in significant improvement, despite there being few randomized controlled trials to reach definitive conclusions, and they concluded that further studies are needed. Recently, a multicentre observational study with GateKeeper™ by Ratto *et al*. 9 has been published. The authors analysed 54 patients with FI, 48 (89%) with no IAS lesion and six (11%) with a defect of IAS not exceeding 60°. After a 12‐month follow‐up, 56% of patients showed an improvement in FI of 75%, and 13% became completely continent.

In this report we included patients with sphincter injury (IAS, EAS or both), even though the circumferential and longitudinal extent of the sphincter defect can reduce the success rate of the procedure. The prostheses were placed in the same position regardless of the position of the defect. Previous studies with GateKeeper™ reported placement in sphincter lesions < 120°. In our case series there were no lesions > 120° but as the first preliminary report of a new device we decided to include not only patients with non‐homogeneous IAS or EAS but also those with IAS and/or EAS defects.

Recently, it has been suggested that the improvement with SphinKeeper™ (THD SpA) may be because of the bulking effect due to its larger size and to the greater number of prostheses placed (from eight to 12). Although results are awaited, at present the only report available is on the feasibility of the procedure 4. Our preliminary report is the first study in the literature that compares preoperative and postoperative results using the CCFIS and the FIQL score; despite a short‐term follow‐up of 6 months and a small sample size, some observations can be made. Validated tests – CCIFS and FIQL score – offer more objective results than the number of FI episodes reported by patients. However, we decided to include episodes of FI per week as a non‐validated parameter, as patients’ perception of FI and the actual episodes reported do not always match. Despite an improvement of the CCFIS and a reduction of FI episodes per week, patients do not necessarily report an improvement of their lifestyle. This could be due to their long history of FI that may profoundly have changed their perception of the condition. Perhaps a longer follow‐up of these patients would clarify the real advantage or disadvantage of the procedure to quality of life.

It is difficult to establish which patients are most suitable for this device. We thought that the inclusion and exclusion criteria used for GateKeeper™ should be the initial criteria for SphinKeeper™ placement. Along with the only other study on SphinKeeper™ 4, no intra‐operative nor postoperative complications were reported in our study. However, despite not having seen any infectious complications, we believe that pre‐implantation antibiotic prophylaxis should be dispensed, following the same strategy as used for other procedures in which a foreign body is implanted. The CCFIS and the total number of FI episodes per week were statistically significantly improved, although no statistical differences were observed in the preoperative and postoperative FIQL score, which is probably due to the small sample size reducing the power of the study.

Patients with lower preoperative CCFIS appeared to have a greater improvement than those with a higher preoperative CCFIS. Higher CCFIS may be associated with more severe incontinence that may require more invasive treatment. If this finding is confirmed by randomized studies with a larger number of cases, it could identify a category of patients that may benefit the most from the SphinKeeper procedure. We reported an improvement in resting pressure on AM, while no significant improvement in squeeze pressure was observed. In the present study, 11 sphincter defects were seen; SphinKeeper™ had no effect on sphincter training, so the degree of sphincter injury may affect surgical outcome.

In our series, we have reported two prosthesis extrusions (one anterior and one posterior) and an anterior dislocation in a patient after 6 months. According to de la Portilla *et al*. 10, the displacement may be due to the location of the implants in a virtual space (the intersphincteric space) and stretching during defaecation may facilitate their movement. However, we could not explain the late displacement. Perhaps it is because of wrong positioning; further studies with more patients and long‐term follow‐up are needed to clarify this hypothesis. According to our preliminary experience, the anterior displacement in female patients may be due to the rectovaginal septum; this virtual space is more subtle in women and therefore more prone to tension within the space; further studies are needed to evaluate this theory. We have not observed any connection between prosthetic dislodgement and functional improvement evaluated with CCFIS, however. The effect of migration or extrusion during clinical follow‐up could better be investigated in larger studies with longer follow‐up.

Despite the promising results, this study has several limitations. First, the small sample size can be a confounding factor and can reduce the power of the analysis and underestimate the complications associated with the use of SphinKeeper™ and be prone to a type I error. However, this is the first paper about this novel procedure with a homogeneous 6‐month follow‐up and includes patients undergoing SphinKeeper™ with sphincter injury. Also, we prescribed dietary fibre supplementation postoperatively to avoid constipation and hard stools that may lead to early displacement. This stool bulking may hide the real benefits of SphinKeeper™, with any advantage not being due to this novel procedure. Furthermore, all patients must now be subjected to long‐term monitoring of the implants, perhaps assisted by three‐dimensional EAUS, that can provide a better assessment and more accurate information on the position and possible displacement of the prostheses. Finally, it is not a randomized trial and has limited follow‐up. For a better assessment of the functional outcome, a prospective randomized study with more patients and with long‐term follow‐up may be required. If these results are confirmed by such studies it may be considered as a less invasive first‐line treatment of FI.

This technique provides the possibility of reconstituting the shape of the anal canal and reinforces the area of scarring by improving the continence contribution from the remaining sphincters. It could be an adjuvant treatment after other procedures for FI.

## Author contributions

La Torre M: acquisition data. Lisi G: interpretation of data, statistical analysis, critical review, drafting and manuscript preparation. Campanelli M: data repository. Milito G: supervising. Clementi I: acquisition of data.
